# Best-practice IgM- and IgA-enriched immunoglobulin use in patients with sepsis

**DOI:** 10.1186/s13613-020-00740-1

**Published:** 2020-10-07

**Authors:** Axel Nierhaus, Giorgio Berlot, Detlef Kindgen-Milles, Eckhard Müller, Massimo Girardis

**Affiliations:** 1 University Medical Center Hamburg, Hamburg, Germany; 2 Dep. of Intensive Care Medicine, University Medical Center Hamburg-Eppendorf, Martinistr. 52, 20246 Hamburg, Germany; 3 University of Trieste, Trieste, Italy; 4 University Hospital Düsseldorf, Heinrich-Heine University, Düsseldorf, Germany; 5 Evangelical Hospital Herne, Herne, Germany; 6 University of Modena, Modena, Italy

**Keywords:** Immunoglobulin, IgM- and IgA-enriched immunoglobulin, Sepsis, Pentaglobin, Hyperinflammation, Immunosuppression

## Abstract

**Background:**

Sepsis is a life-threatening organ dysfunction caused by a dysregulated host response to infection. Despite treatment being in line with current guidelines, mortality remains high in those with septic shock. Intravenous immunoglobulins represent a promising therapy to modulate both the pro- and anti-inflammatory processes and can contribute to the elimination of pathogens. In this context, there is evidence of the benefits of immunoglobulin M (IgM)- and immunoglobulin A (IgA)-enriched immunoglobulin therapy for sepsis. This manuscript aims to summarize current relevant data to provide expert opinions on best practice for the use of an IgM- and IgA-enriched immunoglobulin (Pentaglobin) in adult patients with sepsis.

**Main text:**

Sepsis patients with hyperinflammation and patients with immunosuppression may benefit most from treatment with IgM- and IgA-enriched immunoglobulin (Pentaglobin). Patients with hyperinflammation present with phenotypes that manifest throughout the body, whilst the clinical characteristics of immunosuppression are less clear. Potential biomarkers for hyperinflammation include elevated procalcitonin, interleukin-6, endotoxin activity and C-reactive protein, although thresholds for these are not well-defined. Convenient biomarkers for identifying patients in a stage of immune-paralysis are still matter of debate, though human leukocyte antigen–antigen D related expression on monocytes, lymphocyte count and viral reactivation have been proposed. The timing of treatment is potentially more critical for treatment efficacy in patients with hyperinflammation compared with patients who are in an immunosuppressed stage. Due to the lack of evidence, definitive dosage recommendations for either population cannot be made, though we suggest that patients with hyperinflammation should receive an initial bolus at a rate of up to 0.6 mL (30 mg)/kg/h for 6 h followed by a continuous maintenance rate of 0.2 mL (10 mg)/kg/hour for ≥ 72 h (total dose ≥ 0.9 g/kg). For immunosuppressed patients, dosage is more conservative (0.2 mL [10 mg]/kg/h) for ≥ 72 h, without an initial bolus (total dose ≥ 0.72 g/kg).

**Conclusions:**

Two distinct populations that may benefit most from Pentaglobin therapy are described in this review. However, further clinical evidence is required to strengthen support for the recommendations given here regarding timing, duration and dosage of treatment.

## Background

Sepsis is a global issue which affects an estimated 49 million people every year, potentially leading to 11 million deaths [1]. It is a clinical syndrome in which profound physiological and biochemical changes often lead to a fatal outcome of an infection; the Third International Consensus (Sepsis-3) defined sepsis as a life-threatening organ dysfunction caused by a dysregulated host response to infection. Even after many years of intensive clinical and laboratory research, there is still no specific therapy for sepsis. A subset of sepsis known as septic shock is characterized by profound circulatory, cellular and metabolic abnormalities that are associated with a greater risk of mortality than sepsis alone; with hospital mortality rates > 50% [2, 3].

### Immune pathophysiology of sepsis

**Fig. 1:**
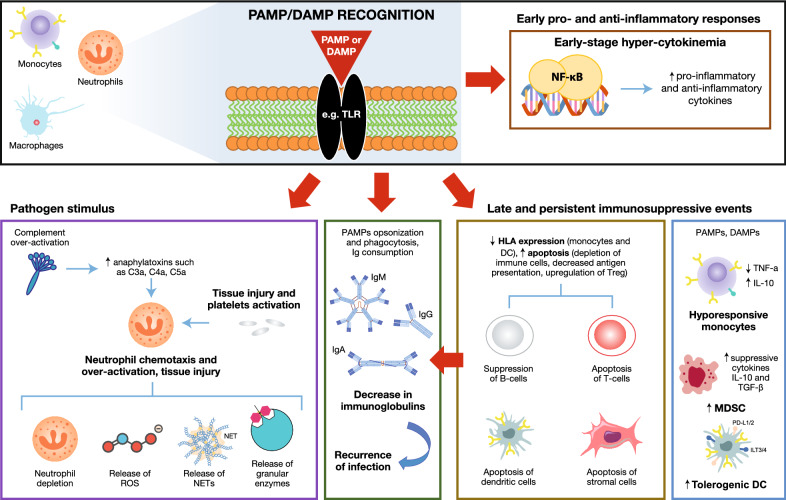
Immune pathophysiology of sepsis. *DAMP* damage-associated molecular pattern, *DC* dendritic cell, *HLA* human leukocyte antigen, *IgM/G/A* immunoglobulin M/G/A, *IL* interleukin, *MDSC* myeloid-derived suppressor cell, *NET* neutrophil extracellular trap, *NF-kB* nuclear factor kappa-light-chain-enhancer of activated B cells, *PAMP* pathogen-associated molecular pattern, *PD-1* programmed death protein 1, *PD-L1* programmed death ligand 1, *ROS* reactive oxygen species, *TGF-β* transforming growth factor β, *TLR* toll-like receptor, *TNF-α* tumor necrosis factor α, *Treg* regulatory T cell

Sepsis is differentiated from uncomplicated infection due to a dysregulated host response to infection. The clinical syndrome of sepsis is initiated by the activation of multiple signaling pathways following the recognition of pathogen-derived molecules [pathogen-associated molecular patterns (PAMPs) e.g. endo- and exotoxins, DNA, lipids] and endogenous host-derived danger signals (damage-associated molecular patterns [DAMPs]) by specific cell-surface receptors on macrophages [toll-like receptors (TLRs)] [4]. Consequently, this leads to the expression of genes involved in inflammation, adaptive immunity, and cellular metabolism [5]. During the course of sepsis, patients often present with multiple features of immunological alterations including systemic inflammatory responses, complement consumption, defects in neutrophil-mediated immunity and decreased serum levels of immunoglobulins finally causing immunosuppression (Fig. 1) [5, 6].

#### Early stage hypercytokinemia

Activation of the TLRs on macrophages such as monocytes and neutrophils induces signal transduction and translocation of nuclear factor kappa-light-chain-enhancer of activated B cells (NF-κB) to the nucleus. NF-κB induces the expression of early activation genes, including inflammatory cytokines such as tumor necrosis factor α (TNF-α), interleukin (IL)-1, IL-12, IL-18 and interferons (IFNs), which further initiate a cascade of other inflammatory cytokines (including IL-6, IL-8, IFN-γ), as well as the suppression of adaptive immunity components [5]. Therefore, in the early stages of sepsis, an increase in the presence of both proinflammatory and anti-inflammatory cytokines is observed at diagnosis [7–9].

#### Effects of complement activation and neutrophil-mediated immunity

In sepsis, there is considerable evidence of complement activation, as reflected by the appearance of complement activation products (anaphylatoxins such as C3a, C4a, C5a) in plasma [10]. Normally, C5a has a beneficial effect and is linked to the recruitment of neutrophils to the site of infection. C5a binding to the C5a receptor (C5aR) transforms the neutrophil into a migratory cell able to invade inflammatory tissue sites and clear pathogens and debris [11]. PAMPs and DAMPs induce oxidative burst leading to the release of reactive oxygen species and granular enzymes, and release neutrophil extracellular traps (NETs). Excessive activation of C5a in the development of sepsis is linked to several processes including apoptosis of lymphocytes, aggravation of systemic inflammation and neutrophil dysfunction [12]. Excessive C5a leads to down-regulation of C5aR during sepsis and can have detrimental effects resulting in homing of neutrophils to the microvasculature, inflammation, tissue damage, thrombosis and multiple organ failure. Blockage of C5a or C5aR inhibits the development of sepsis in mouse models, whereas in patients with sepsis, a downregulated C5aR and high C5a levels correlate with poor prognosis [13].

#### Decreased levels of immunoglobulins

There have been several observations of decreased immunoglobulins among patients at sepsis diagnosis, in particular decreased levels of the three major immunoglobulin isotypes, immunoglobulins G, M and A (IgG, IgM and IgA, respectively; Table 1. [14–22]). A synergistic role of IgG, IgM and IgA in sepsis and septic shock has been described [21, 24], and the combined presence of low levels of endogenous IgG, IgM and IgA in plasma is associated with reduced survival in patients with severe sepsis or septic shock [21, 25]. The mechanism or the underlying cause for low levels of immunoglobins in sepsis are not entirely clear, but it has been suggested that it may be due to their reduced production/secretion due to immunosuppression, vascular leakage secondary to endothelial dysfunction, redistribution into inflamed tissues, over-utilization by the complement system and excessive catabolism [6, 21, 22, 26, 27].

**Table 1:** Studies reporting on immunoglobulin levels and kinetics in patients with sepsis

References	Study objective	Study design/enrolled patients	Immunoglobulin findings	Outcomes
Taccone et al. [14]	Evaluate the time course of gamma-globulin concentrations in patients with septic shock, to define the frequency of low immunoglobulin concentrations, and to investigate the relationship of immunoglobulin concentrations to disease severity and outcome	Prospective observational study 21 patients (aged ≥ 18 years old) with community-acquired septic shock	76% of patients (16/21) had hypo-gammaglobulinemia (single or combined immunoglobulin deficiency) at admission: 7 patients had isolated low IgG concentrations, 4 patients had isolated low IgM concentrations, and 3 patients had low IgG and IgM concentrations Two patients had low concentrations of IgG, IgM, and IgA and died with refractory shock within 2 days Patients with low IgG concentration on Day 1 had persistent low levels throughout the ICU stay. Almost all patients with normal IgG levels maintained normal concentrations throughout their stay (1 patient had a transient decrease in IgG on Day 3)	Patients with low IgG concentrations were indistinguishable at baseline from patients with normal IgG concentrations but had fewer vasopressor-free days (P = 0.02) and more frequently developed acute lung injury/acute respiratory distress syndrome (P = 0.02) There was no significant difference in outcomes in patients with normal or low IgM levels All deaths occurred in patients with low IgG concentrations (P = 0.01)
Myrianthefs et al. [15]	Investigate the time course of IgG and IgM concentrations in patients who developed septic shock during their ICU stay	Observational cohort study 38 patients who developed septic shock during their ICU stay	45% of patients (17/38) had hypo-gammaglobulinemia (single or combined immunoglobulin deficiency) on admission: 7 patients had isolated low IgG levels, 5 patients had isolated low IgM concentrations, and 5 patients had low IgG and IgM levels Low levels of IgG were resolved within 10 days in the 5 patients who survived in the group with low IgG IgM concentrations improved over time in patients with and without low IgG levels	There were no significant differences regarding length of ICU or hospital stay, oxygenation index (PaO_2_/FIO_2_), duration of vasopressor use, or duration of mechanical ventilation in those with low or normal IgG levels No comparative analyses were provided for patients with low or normal IgM levels
Andaluz-Ojeda et al. [16]	Evaluate the quantitative changes in the status of immunocompetence in severe sepsis over time and its potential influence on clinical outcome	Prospective observational cohort study 50 patients (aged ≥ 18 years old) with severe sepsis or septic shock	Survivors exhibited a progressive increase in IgG, IgA, and IgM levels from Day 1 to Day 10	Compared to survivors, septic patients who did not survive had significantly lower levels of IgG in the first 24 h following admission to the ICU There was no significant difference in IgA or IgM levels between survivors and non-survivors
Venet et al. [17]	Measure the endogenous levels of circulating IgG, IgA, and IgM in a cohort of septic shock patients	Prospective observational cohort study 62 consecutive patients (aged ≥ 18 years old) with septic shock	At Days 1–2, 61%, 40%, and 9% of patients had IgG, IgM, and IgA concentrations below the lowest limit of age-matched reference values, respectively Circulating IgG and IgM concentrations increased over time, by Days 5–7, 61% of patients had IgG and IgM levels within the range of normal values	Changes in immunoglobulin levels did not appear to be associated with increased mortality, morbidity, or severity after septic shock Reduced immunoglobulin level was correlated with reduced protein concentrations at Days 1–4 suggesting an apparent hypogammaglobulinemia is present during this time period in septic shock patients
Tamayo et al. [18]	Investigate the relationship between endogenously produced immunoglobulins and the clinical outcome in septic shock	Retrospective study 42 patients with septic shock and 36 patients with systemic inflammatory response syndrome	Both patients with systemic inflammatory response syndrome and septic shock showed subnormal levels of total IgG, IgG2, and IgM	Patients with septic shock who died showed the lowest levels of total IgG and IgG1 Univariate Cox regression analysis showed that levels of IgG1, IgG2, IgG3, IgM, IgA, and total IgG were inversely associated to the probability of death at 28 days Multivariate analysis showed that IgG1, total IgG, IgM, and IgA behaved as independent protective factors against mortality (HR, P): 0.23, 0.026; 0.16, 0.028; 0.11, 0.042; 0.05, 0.010, respectively
Giamarellos-Bourboulis et al. [19]	Investigate the kinetics of IgM during the different stages of sepsis	Prospective observational multicenter cohort study 332 critically ill patients were enrolled	Serum IgM was decreased in septic shock compared to patients with systemic inflammatory response syndrome and patients with severe sepsis Paired comparisons at distinct time points of the sepsis course showed that IgM was decreased only when patients deteriorated from severe sepsis to septic shock	Serial measurements in patients who progressed from severe sepsis to septic shock, beginning from the early start of vasopressors, showed that the distribution of IgM over time was significantly greater for survivors than for non-survivors
Průcha et al. [20]	Assess the frequency of hypogammaglobulinemia in patients with systemic inflammatory response syndrome, severe sepsis, and septic shock	Retrospective study 708 patients with systemic inflammatory response syndrome, severe sepsis, and septic shock	IgG, IgA, and IgM hypogammaglobulinemia was demonstrated in 25%, 3%, and 12% of patients with severe sepsis, and 24%, 2%, and 13% of septic shock patients, respectively	Mortality in patients with severe sepsis or septic shock and IgG hypogammaglobulinemia was significantly higher than in those with normal IgG levels Mortality in patients with septic shock and IgM hypogammaglobulinemia was significantly higher than in those with normal IgM levels. In patients with severe sepsis, no significant difference in mortality was observed
Bermejo-Martín et al. [21]	Evaluate the association between immunoglobulin levels in plasma and survival in patients with severe sepsis	Prospective observational multicenter cohort study 172 patients (aged > 18 years old) admitted to the ICU with severe sepsis/septic shock	At time of diagnosis, 27.9%, 39.2%, and 19.2% of patients had immunoglobulin concentrations below the normal reference values for IgG1, IgM, and IgA, respectively	Kaplan–Meier analysis showed that levels below normal reference values for IgG1, IgM, and IgA were associated with shorter survival times Multivariate regression analysis showed that low levels of IgG1 were a risk factor for mortality (OR: 2.50, 95% CI 1.04–6.03; P = 0.042) The combined presence of IgG1, IgM, and IgA levels below the normal threshold had a synergistic impact on mortality risk (OR: 5.27, 95% CI 1.41–19.69; P = 0.013). A similar effect was observed for combined low levels of IgG1 and IgA:,and IgG1 and IgM
Shankar-Hari et al. [22]	Evaluate the additional mortality risk associated with subnormal IgG concentrations in adults with sepsis managed in an ICU setting	Systematic review of 8 studies 438 adult patients with sepsis	IgG concentrations increased over time in most studies	Subnormal IgG levels on the day of sepsis diagnosis did not increase the risk of death in adult patients with severe sepsis and/or septic shock by both fixed effect and random effect meta-analysis (M-H pooled OR: 1.32 [95% CI 0.93–1.87] and D + L pooled OR: 1.48 [95% CI 0.78–2.81], respectively)
Tian et al. [23]	Study the relationship between circulating B cells and plasma IgM levels and sepsis survival rate	Systematic review and meta-analysis of 11 studies 829 patients (aged > 18 years old) with sepsis and/or septic shock	Plasma IgM level was significantly decreased in septic patients (SMD = − 2.35, 95% CI − 2.94, − 1.76; P < 0.00001, I^2^ = 0%) compared with healthy controls Plasma IgM level was significantly lower in sepsis survivors versus sepsis non-survivors (SMD = − 0.31, 95% CI − 0.53, − 0.09; P = 0.005, I^2^ = 50%)	The reduction of circulating B cells and IgM plasma levels is negatively correlated with sepsis survival

Previous studies reporting on immunoglobulin levels and kinetics in patients with sepsis were identified by searching PubMed using the following search terms: (“immunoglobulin”[Title/Abstract] AND (“level”[Title/Abstract] OR “kinetic”[Title/Abstract])) AND (“sepsis”[Title/Abstract] OR “septic”[Title/Abstract]). The results were filtered for English language and visually assessed for relevance. Studies which did not report on the levels or kinetics of immunoglobulins in adult patients with sepsis were omitted. Additional studies were identified from reference lists of included studies

*CI* confidence interval, *HR* hazard ratio, *ICU* intensive care unit, *IgA* immunoglobulin A, *IgG* immunoglobulin G, *IgM* immunoglobulin M, *M-H* Mantel–Haenszel, *OR* odds ratio, *SMD* standard mean difference

#### Late immunosuppressive events

In sepsis, increased circulating levels of myeloid-derived suppressor cells (MDSCs) have been observed; these cells secrete multiple anti-inflammatory cytokines, including IL-10 and transforming growth factor-β (TGF β), which suppress immune function [5, 28]. In addition, an apoptotic decrease in antigen-presenting dendritic cells and monocytes has been observed, along with a loss of their proinflammatory cytokine production [29–33]. Human leukocyte antigen–antigen D related (HLA-DR) expression on monocytes and dendritic cells is also downregulated, which decreases responsiveness, and the failure of monocytes to recover HLA-DR levels predicts a poor outcome from sepsis [34].

Natural killer-cell, B- and T-lymphocyte depletion can also be observed in peripheral blood along with an increase in apoptosis of dendritic cells (antigen-presenting cells [APCs]) and stromal cells [35–40]. In the course of sepsis, inhibitory immune checkpoint molecules, including programmed death protein 1 (PD-1), are upregulated on T cells, APCs or peripheral tissue epithelial cells. These molecules regulate leukocyte functions, leading to immune cell apoptosis (contributing to T cell exhaustion), APC dysfunction and expansion of regulatory T (Treg) cells [5, 39, 41–44]. Although cell death in innate and adaptive immunity is initially beneficial to the host, by downregulating the inflammatory responses in sepsis, the extensive loss of immune cells may compromise the ability of the host to further eliminate invading pathogens. It has been shown that preventing immune cell apoptosis markedly improved survival [45].

### Why focus on immunoglobulins?

Polyvalent intravenous immunoglobulins, within the network of inflammation and immunity, represent a promising approach to modulate both the pro- and anti-inflammatory processes [46]. However, studies have observed that polyclonal immunoglobulin formulations containing only IgG do not result in improved mortality rates in patients with sepsis [47–49]. On the other hand, although the underlying mechanisms for IgM- and IgA-enriched immunoglobulins to exert beneficial effects in patients with severe sepsis and septic shock is not completely understood, systematic reviews have generally concluded that IgM- and IgA-enriched immunoglobulin preparations are associated with a reduction in mortality [50, 51]. A recent meta-analysis, with trial sequential analysis that included 19 studies involving a total of 1530 patients, found that mortality was significantly reduced in the IgM- and IgA-enriched immunoglobulin group compared with the control group [52].

Currently, the commercially available IgM- and IgA-enriched immunoglobulin formulation is Pentaglobin (12% IgM, 12% IgA and 76% IgG). A different preparation, trimodulin (approximately 23% IgM, 21% IgA and 56% IgG), is in clinical development [53]. The data on the efficacy and safety of IgM- and IgA-enriched immunoglobulin therapy in patients with sepsis therefore comes from the use of Pentaglobin (Table 2, [54–74]).

**Table 2:** Studies reporting on the outcomes of IgM and IgA-enriched immunoglobin therapy

References	Study design/enrolled patients	Cumulative dose	Outcome
Just et al. [54]	Prospective, randomised, controlled clinical trial 104 intensive care patients (50 patients in treatment group, 54 patients in control group)	Pentaglobin: initially 5 g, then 5 g every 12 h for 36 h (total 20 g) combined with antibiotics	There was a significant decrease in recovery time, ventilation time, and time spent in the ICU in the treatment group compared to the control group
Vogel [55]	Prospective, randomized, controlled study 50 patients with sepsis (25 patients in treatment group, 25 patients in control group)	Pentaglobin: 10 g/day for 3 days	There was a ~ 20% lower mortality rate in patients receiving Pentaglobin compared with the control group
Wesoly et al. [56]	Prospective, randomized, controlled study 35 patients with septic postoperative complications (18 patients in treatment group, 17 patients in control group)	Pentaglobin: 250 mg/kg/day	Endotoxin titers decreased, along with a reduction in mortality and shortening of hospitalization and mechanical ventilation time, in patients receiving Pentaglobin compared with control
Schedel et al. [57]	Prospective, randomized, controlled clinical trial 55 patients with gram-negative septic shock (27 patients in treatment group, 28 patients in control group)	Pentaglobin: for 3 days according to the following schedule: Day 1: 30 g over > 8 h; Days 2 and 3: 15 g over > 8 h	Patients treated with Pentaglobin had a significantly lower rate of sepsis-related mortality compared to the control group
Behre et al. [58]	Prospective pilot study and randomized, controlled trial Pilot study: 21 patients with acute leukemia or non-Hodgkin’s lymphoma and sepsis syndrome Randomized controlled trial: 52 patients with hematological malignancies and sepsis syndrome (30 patients in treatment group, 22 patients in control group)	Pentaglobin: Initial bolus of 10 g followed by 5 g every 6 h for 3 days	Patients treated with Pentaglobin had a significantly lower rate of all-cause 28-day mortality compared with those who received 5% human albumin
Rodríguez et al. [59]	Multicenter, prospective, randomized, double-blind clinical trial 37 patients with abdominal sepsis (20 patients in treatment group, 17 patients in control group)	Pentaglobin: 350 mg/kg/day for 5 days	There was no significant difference in organ dysfunction, organ failure, or mortality between the patients receiving Pentaglobin and the control group. The mortality rate was lower in the Pentaglobin versus control group without reaching statistical significance
Reith and Mittelkötter [60]	Prospective, controlled trial 67 patients with severe sepsis or septic shock (35 patients in treatment group, 32 patients in control group)	Pentaglobin: 15-20 g/day for 3 days	Patients treated with Pentaglobin had a significantly lower mortality rate compared with patients in the control group
Tugrul et al. [61]	Prospective, randomized, controlled study 42 patients with severe sepsis (21 patients in treatment group, 21 patients in control group)	Pentaglobin: 250 mg/kg/day over 6 h for 3 days	There was no significant difference in organ morbidity, septic shock incidence, or mortality between the treatment and control groups
Karatzas et al. [62]	Prospective, randomized, controlled study 68 patients with severe sepsis (34 patients in treatment group, 34 patients in control group)	Pentaglobin: 250 mg/kg/day over 6 h for 3 days	Patients treated with Pentaglobin had a significantly lower rate of 28-day mortality compared to the control group
Reith et al. [63]	Prospective, randomized controlled study 64 patients with abdominal infection (31 patients in treatment group, 33 patients in control group)	Pentaglobin: 10 g within 6 h of surgery followed by 55 g over the next 66 h by continuous perfusion (total: 1300 mL over 3 days)	There was no significant difference in incidence of fever, percentage of days with fever, mean body temperature, or duration of stay in hospital between those receiving Pentaglobin or albumin
Rodríguez et al. [64]	Prospective, randomized, double-blind controlled study 56 patients with severe sepsis and septic shock of intra-abdominal origin (29 patients in treatment group, 27 patients in control group)	Pentaglobin: 350 mg/kg/day for 5 days	There was a ~ 20% reduction in mortality rate in patients receiving Pentaglobin compared with the control group; however, there was no significant difference in organ dysfunction, organ failure, or mortality between the 2 groups
Buda et al. [65]	Retrospective case-controlled study 66 patients diagnosed with sepsis after cardiac surgery (22 patients in treatment group, 44 patients in control group)	Pentaglobin: 250 mg/kg daily for 3 days	Pentaglobin did not significantly reduce mortality in the overall study population. However, in the subgroup of patients with severe sepsis, it improved the survival rate significantly
Hentrich et al. [66]	Multicenter, prospective, randomized, controlled study 206 neutropenic patients with sepsis syndrome or septic shock after receiving chemotherapy for severe hematologic disorders (103 patients in treatment group, 103 patients in control group)	Pentaglobin: 65 g over 3 days according to the following schedule: 10 g initially (0.5 mL/min) followed by 11 infusions of 5 g, repeated every 6 h	There was no significant difference in all-cause 28- or 60-day mortality, or sepsis-related 28-day mortality between patients receiving Pentaglobin or human albumin
Yavuz et al. [67]	Retrospective study 118 patients with sepsis-induced multiple organ dysfunction syndrome (56 patients in treatment group, 62 patients in control group)	Pentaglobin: 250 mg/kg/day for 3 days	Patients who received IgM-enriched immunoglobulins had significantly lower overall mortality and 28-day case fatality rates and a shorter length of ICU stay compared with the control group
Toth et al. [68]	Prospective, randomized, controlled pilot study 33 patients with early septic shock accompanied by severe respiratory failure (16 patients in treatment group, 17 patients in placebo group)	Pentaglobin: 250 mg/kg over 8 h for 3 days	There was no significant difference in organ dysfunction between patients who received Pentaglobin and placebo
Brunner et al. [69]	Prospective, randomized, double-blind, placebo-controlled trial 38 critically ill patients with multiple organ failure, systemic inflammatory response syndrome, and early clinical signs of critical illness polyneuropathy and/or myopathy (19 patients in treatment group, 19 patients in placebo group)	Pentaglobin: 250 mg/kg body weight/day as a continuous intravenous infusion at a rate of 2 g/h for 3 days	Early treatment with Pentaglobin did not significantly improve critical illness polyneuropathy and/or myopathy or influence length of ICU stay or mortality in critically ill patients
Cavazzuti et al. [70]	Retrospective cohort study 168 patients with septic shock (92 patients in treatment group, 76 patients in control group)	Pentaglobin: 250 mg/kg/day (20 mg/kg/h) for 3 days	Early adjunctive treatment with IgM-enriched immunoglobulins resulted in an approximately 20% reduction in the absolute risk of 30-day mortality in patients with septic shock
Giamarellos-Bourboulis et al. [71]	Retrospective analysis 200 patients with confirmed severe sepsis or septic shock caused by nosocomial multi-drug resistant Gram-negative bacteria infection (100 patients in treatment group, 100 in control group)	Pentaglobin: Mean daily dose: 30 g/day administered as a 5–6-hour continuous infusion for 5 days	Patients treated with Pentaglobin had a significantly lower rate of all-cause 28-day mortality compared with the control group
Berlot et al. [72]	Retrospective single-center study 355 patients with septic shock	Pentaglobin: 250 mg/kg/day over 10 h for 3 days (total dose 750 mg/kg)	Earlier administration of Pentaglobin was associated with a decreased risk of in-ICU mortality, both in patients with septic shock caused by any pathogens and in patients with MDR-related septic shock
Willuweit et al. [73]	Retrospective study 21 patients with sepsis-related vasoplegia post-liver transplant	Pentaglobin: 250 mg/kg over 12 h for 3 days	Patients who received IgM-enriched immunoglobulins had significantly decreased levels of inflammatory markers and a reduction in vasopressors required to maintain hemodynamic stability 30-day mortality was 14.3%, significantly less than calculated mortality (greater than 90%) based on Sepsis-Related Organ Failure Assessment scores
Domizi et al. [74]	Single-center, randomized, double-blind, placebo-controlled Phase 2 trial 20 patients diagnosed with sepsis or septic shock for less than 24 h (10 patients in the treatment group, 10 patients in the control group)	Pentaglobin: 250 mg/kg (5 mL/kg)/day for 3 days	A 72-hour infusion of Pentaglobin in patients with sepsis or septic shock was associated with an increase in sublingual microvascular perfusion

Previous studies reporting on the outcomes of Pentaglobin therapy were identified by supplementing a recent publication which systematically searched PubMed, Cochrane Library, ISI Web of Knowledge, and Embase databases to update the 2013 edition of the Cochrane review from inception to June 2018 [52]. Their search strategy consisted of: [iviggma (All Fields) OR [igm (All Fields) AND enriched (All Fields)] OR [pentaglobulin (Supplementary Concept) OR pentaglobulin (All Fields) OR pentaglobin (All Fields)] AND [sepsis (MeSH Terms) OR sepsis (All Fields)]. We used the same search category in PubMed to complete the search from June 2018 to June 2020

*ICU* intensive care unit, *IgA* immunoglobulin A, *IgG* immunoglobulin G, *IgM* immunoglobulin M, *MDR* multidrug-resistant

Relevant mechanisms of action of IgM- and IgA-enriched immunoglobulins include opsonization and phagocytosis of causal pathogens [75], neutralization of virulence factors including bacterial endo- and exotoxins [76, 77], as well as immunomodulation via interaction with complement factors [78, 79] and prevention of hyper-inflammatory responses. Immunoglobulins have also been shown to downregulate IL-2 production, resulting in a significant inhibition of human T-lymphocyte alloproliferative response in vitro as well as in lectin-stimulated peripheral blood mononuclear cells [80]. However, in addition to a modulation of IL-2, IgM and IgA enriched immunoglobulin exhibited differential effects on the release of pro-inflammatory cytokines (IFN-γ, TNF-α and IL-6) during mixed lymphocyte reaction response [80]. Additionally, in vitro and in vivo models have shown an upregulation of IL-10 following IgM and IgA enriched immunoglobulin administration [81, 82]. Furthermore, a recent clinical study in patients treated with either IgM and IgA enriched immunoglobulin or placebo (NaCl) showed a significant decrease of IL-6 and IL-10 levels at 72 h in the IgM and IgA enriched immunoglobulin group only [74]. Ex vivo data also showed that the investigational preparation, trimodulin, lowered monocyte expression of recognition receptors (TLR2 and TLR4) and coagulation receptors (CD11b and CD64) and also reduced lymphocyte proliferation and release of pro- and anti-inflammatory cytokines including TNF-α and IL-10 [83]. Recently, a beneficial effect of IgM administration on microvascular perfusion parameters could be demonstrated in humans [74], which corroborated earlier research in an animal model of endotoxemia [84]. These effects are in line with positive effects of IgM on septic encephalopathy and the integrity of the function of the blood–brain barrier [85, 86].

The benefits of IgM and IgA enriched immunoglobulin have been gathered from different studies with clinically heterogeneous patients, a wide variety of treatment protocols (e.g. dosage) and in settings with variable access to laboratory diagnostics [87]. Understanding which patients may benefit most from Pentaglobin therapy is of high clinical relevance given the need for a balance between a potential reduction in mortality as well as the relatively high cost and availability of treatment. A previous publication sought to provide guidance on optimal IgM- and IgA-enriched immunoglobulin use [88], however, in the intervening years, further clinical data have been generated and more clinical experience has been gathered to warrant an update to this publication. Furthermore, there is increasing interest in the need for ‘personalized medicine’ [89]. Previous immunomodulatory trials in sepsis have often failed in part due to a failure to correctly identify the appropriate target group [45, 90–93]. Therefore, identification of the appropriate target population for IgM- and IgA-enriched immunoglobulin therapy and tailoring an intervention accordingly could be of great benefit.

However, current international guidelines for the management of sepsis and septic shock from the Surviving Sepsis Campaign advise against the use of intravenous immunoglobulins (IVIGs) in these conditions [94]. This recommendation was graded as weak, with low quality of evidence, and was based largely on a Cochrane meta-analyses which predominantly included relatively small trials performed with IgG. The only large study included used IgG and showed no effect [47].

With this in mind, and given the relatively new concept of sepsis being a ‘dysregulated’ host/immune response, as well as how excessive consumption and insufficient production of immunoglobulins could result in (acquired) deficiency, an expert meeting was organized in March 2019 in Brussels, Belgium during the 39th International Symposium on Intensive Care and Emergency Medicine (ISICEM) congress (Additional file 1: Appendix S1). This working group consisted of six experienced academic critical care physicians from Italy, Germany and Hungary, who had more than a decade of both scientific and clinical experience using immunoglobulins in the context of adjunctive sepsis therapy. The participants discussed which septic patients most benefit from IgM- and IgA-enriched immunoglobulins, current best practice management in different patient populations and how the sepsis treatment landscape has changed over recent years. A consensus report was produced from this expert meeting, which formed the basis of this manuscript, and literature searches using the relevant databases were carried out to identify further evidence of the topics discussed. Additional references were then included during the preparation of the manuscript.

## Which patients may benefit most from IgM- and IgA-enriched immunoglobulin therapy?

### Defining patient phenotypes

Sepsis is a complex syndrome shaped by pathogen and host factors with specific characteristics that progress over time [2] and a ‘one size fits all’ approach to treatment with IgM- and IgA-enriched immunoglobulins seems inappropriate. We have identified two distinct patient groups who may benefit most from treatment with IgM and IgA enriched immunoglobulin, which can be defined as: (1) those with an acute disease onset, who are heavily inflamed, showing signs of imminent or overt septic shock (patients in a hyperinflammatory stage); and (2) those with an immunocompromised phenotype, often with a long-term intensive care unit (ICU) stay and a higher incidence of viral reactivation and/or nosocomial infections (patients in an immunosuppressive stage). Two-thirds of patients who have combatted initial sepsis may suffer from persistent hyperinflammation, elevated immunosuppression biomarkers and catabolism syndrome developing ‘persistent critical illness’ while still on the ICU; these patients often experience poor long-term outcomes such as high 1-year mortality rates and are frequently disabled by cognitive dysfunction, neuromyopathies, immunological dysfunction and other complications [95].

It must be noted that evidence for the clinical phenotypes and management for these two patient populations can be variable and recommendations made in this review are, therefore, based on both published evidence and the authors’ clinical experience.

### Patients with hyperinflammation

#### Clinical phenotype

##### Scientific evidence

There are several clinical consequences of hyperinflammation, which affect almost all organs in the body and result in a marked elevation of many biomarkers such as procalcitonin [PCT], IL-6 and C-reactive protein [CRP] [2, 96]. A post hoc analysis of a randomized, controlled study in patients with severe community-acquired pneumonia and elevated baseline CRP, reduced IgM or both, showed a reduction in mortality rate, ventilation requirements and length of hospital stay with the investigational IgM-preparation trimodulin compared with placebo [53].

Another potential method of identifying patients who may best benefit from IgM- and IgA-enriched immunoglobulin treatment could be the use of an adapted version of the predisposition, insult/infection, response and organ dysfunction (PIRO) score—the Torino (TO)-PIRO score [97]. However, the score has its limitations and requires validation through use in clinical practice and results gathered from large databases.

The shock index is an effective, low-cost, easily available bedside measurement tool for the initial assessment of patients at risk for sepsis; patients who present with a normal shock index (< 0.7) have been found to be at very low risk for severe sepsis [98]. The shock index may also help in the evaluation of fluid resuscitation as well as predict the presence of lactic acidosis, development of organ failure and mortality [99]. According to the international consensus definition, septic shock is defined by a vasopressor requirement to maintain a mean arterial pressure of ≥ 65 mmHg and serum lactate level > 2 mmol/L (> 18 mg/dL) in the absence of hypovolemia [2, 100].

##### Clinical experience

The biomarker thresholds for starting IgM and IgA enriched immunoglobulin therapy have not been well-defined for most of the listed parameters (PCT, IL-6 and CRP). As an example of biomarker-driven interventions, Branche et al. [101] suggest a PCT cut-off of > 0.5 μg/L for antibiotic use. Conversely, rather than threshold values serving as an indicator for starting therapy, observing the kinetics of these biomarkers may better serve to indicate the effectiveness of overall treatment and assist in the determination of the required duration of therapy. Although there is little published evidence with immunoglobulin treatment to support this recommendation [74], there have been studies with ICU patients treated with antibiotics [102, 103].

#### Timing of therapy

##### Scientific evidence

Among ICU patients with septic shock caused by any pathogens [including those that are multidrug-resistant (MDR)], those who received IgM- or IgA-enriched immunoglobulins earlier (median delay 12 h versus 14 h) were more likely to survive than those who received them later [71, 72]. This suggests that the timing of treatment may play a critical role in treatment efficacy and patients with hyperinflammation should be treated with IgM- and IgA-enriched immunoglobulins as soon as possible.

##### Clinical experience

Patients with particularly low IgM levels should be treated as soon as possible; the threshold for low IgM is uncertain, but we suggest ≤ 40–80 mg/dL. Although starting treatment as soon as possible (within 24 h) may lead to overtreatment in some patients, this is felt to outweigh the increased risk of mortality in some patients if treatment is delayed. Given the benefit of early treatment, IgM- and IgA-enriched immunoglobulin administration should be initiated prior to the cause of sepsis/severe infection being identified.

#### Appropriate dosage

##### Current recommendation

The summary of product characteristics (SmPC) currently recommends Pentaglobin therapy at a dose of 5 mL (0.25 g)/kg body weight/day for 3 consecutive days with an infusion rate of 0.4 mL/kg/h, further infusions may be required depending on the clinical course. Dosing depends on the immunological status of the patient and the severity of the disease. A higher dosage (7 mL/kg/day for 5 days) of IgM- and IgA-enriched immunoglobulin was used in a prospective study assessing the impact of adjuvant therapy in combination with antibiotics in patients with abdominal sepsis [64]. Dosing of IgM- and IgA-enriched immunoglobulin is also an important consideration in two ongoing Pentaglobin trials; in one septic shock study (IgM-FAT trial), the dosage based on IgM serum levels is compared with the dosage recommended in the SmPC (NCT04182737). In the randomized controlled PEPPER trial, which is currently recruiting patients, a single-mode continuous infusion of 0.4 mL/kg/hour without initial bolus is administered until a total dose of 7 mL/kg/day has been reached; this administration is repeated for 5 consecutive days (NCT02810704) [104].

##### Clinical experience

Dosing varies between hospitals; however, it may be reasonable to consider an initial bolus since reaching higher IgM levels earlier could be beneficial, i.e. an initial bolus of Pentaglobin at a rate of up to 0.6 mL (30 mg)/kg/h for the first 6 h, followed by a continuous maintenance rate of 0.2 mL (10 mg)/kg/h for 72 h for at least 3 days (total dose ≥ 0.9 g/kg). If possible, IgM levels should be determined upon admittance and monitored regularly. It is not currently known which target values are appropriate to achieve in patients with sepsis (i.e. ‘normal’ or ‘supranormal’), however, the doubling of a patient’s IgM level from the start of treatment has been observed to greatly increase their likelihood of survival. If IgM levels do not increase after 3 days, treatment should be prolonged for at least 2 additional days. In settings where it is not feasible to measure IgM levels regularly, treatment should be started independently from the initial level of IgM. Blood should, however, be drawn at admission prior to treatment and the initial IgM levels may be determined later.

#### Which patients are not eligible for treatment?

Patients ineligible for therapy are those with a standing do not resuscitate (DNR) order or limitation of therapy, incurable metastatic malignant disease or unstable hematological malignancies.

*Recommendations for patients with hyperinflammation*

Clinical phenotype:

- Shock index (abnormal shock index ≥0.7) [98, 99]
- Laboratory evidence of hyperinflammation e.g. high values of PCT, IL-6, CRP [105]
- Septic shock markers: serum lactate [2, 100] and arterial pressure of <65 mmHg
- Clinical examples include meningococcal sepsis, toxic shock syndrome, necrotizing fasciitis and severe community-acquired pneumonia (sCAP) [53, 106, 107]

Timing:

- As early as possible, particularly in those with low IgM levels and high inflammatory load, and within 24 hours [72]

Dosage:

- Total dose of ≥0.9 g/kg
- Rate of 0.6 mL (30 mg)/kg/hour for the first 6 hours followed by a continuous maintenance rate of 0.2 mL (10 mg)/kg/hour for 72 hours *(Expert Opinion)*
- Determine IgM levels if possible; if no increase is observed prolong treatment for at least 2 additional days *(Expert Opinion)*

Exclusion criteria:

- Standing DNR order or limitation of therapy, incurable metastatic malignant disease, unstable hematological malignancies

### Patients with immunosuppression

#### Clinical phenotype

##### Scientific evidence

Our understanding of dysregulated immunity in sepsis has shifted in the last decade. Excessive immune activation has previously been the focus of attention in sepsis; however, more recent evidence has highlighted the important role of immunosuppression (or ‘sepsis-induced immunoparalysis’) as the prevailing immune dysfunction associated with morbidity and mortality [96]. The clinical symptoms/phenotypes of immunosuppression are not as well defined as those of hyperinflammation, though it is recognized that these patients have increased susceptibility to secondary infections [96]. Many patients with septic shock remain in the ICU for weeks with chronic critical illness, and mortality rates increase after 28–30 days following repeated nosocomial infections [108, 109]. These chronic critically ill patients with persistent immunosuppression eventually succumb following viral infections (reactivation and de novo infection) as well as bacterial and fungal infections, and successfully managing and treating these patients is a significant challenge [71, 110–112]. Low HLA-DR expression can also be a marker of immune dysfunction and a predictor of mortality in severe sepsis and septic shock patients [113–115].

In the absence of effective characterization of immune status, nosocomial MDR infection can be considered a surrogate marker for immunosuppression, although this must be considered within the context of local resistance patterns [116]. Cytomegalovirus (CMV) and herpes simplex virus (HSV) reactivation also reflect acquired immunosuppression manifesting as T-cell exhaustion [117]. Measurement of the immune status, such as PD-1/programmed death ligand 1 (PD-L1) expression on T cells and dendritic cells, lymphocyte count, HLA-DR expression on monocytes, immunoglobulin levels and inflammatory markers (e.g. CRP, IL-6 or PCT) are potential diagnostic biomarkers to be considered [105, 118, 119]. Low HLA-DR expression, in particular, may correlate with low lymphocyte counts in the differential blood count and lymphocyte count is also readily available in most hospitals.

##### Clinical experience

There are currently insufficient means to characterize the immune status of a patient on a day-to-day basis, particularly between different centers. Therefore, choosing the most meaningful biomarkers for identifying patients with immune paralysis is still a matter of debate; until now, repeated measurement of HLA-DR expression on monocytes, lymphocyte count and viral reactivation have been proposed as potential biomarkers [119–122]. Measuring IgM level may be of additional benefit in immunocompromised patients, and persistently low IgM levels (≤ 40–80 mg/dL) may prompt substitution. As previously mentioned, however, actionable thresholds for IgM in this patient group are largely elusive and further data are required to confirm this hypothesis. It is also acknowledged that in some settings monitoring IgM levels is not feasible and as yet cannot be considered a mandatory criterion for treatment [123]. Further research and technological development regarding the identification and monitoring of patients with immunosuppression is certainly warranted.

#### Timing of therapy

##### Clinical experience

Providing an exact recommendation on timing of IgM therapy in this population is difficult as the most appropriate data are from patients with severe sepsis or septic shock. However, in our experience, the timing of IgM therapy may be less critical in this phenotype, though it’s largely agreed that patients should be treated early, taking into account that the clinical manifestations of septic shock are more subtle in immunosuppressed patients compared with non-immunosuppressed patients. Either way, the 6-h sepsis bundle should be completed, and the patient should fulfil the clinical criteria for septic shock.

#### Appropriate dosage

##### Current recommendation

The SmPC currently recommends Pentaglobin therapy at a dose of 5 mL (0.25 g)/kg body weight/day for 3 consecutive days with an infusion rate of 0.4 mL/kg/h. Further infusions may be required depending on the clinical course. Dosing depends on the immunological status of the patient and the severity of the disease.

##### Clinical experience

Pentaglobin should be administered with a continuous maintenance rate of about 0.2 mL (10 mg)/kg/h for 72 h (total dose of ≥ 0.72 g/kg), and an initial bolus is not considered beneficial. IgM levels should be determined if possible and if no increase is observed, treatment should be prolonged for at least 2 additional days. Given the lack of supporting evidence and clinical experience in treating this population, we acknowledge that dose and a timeline for immunosuppressed patients with late-onset septic shock have yet to be elucidated.

#### Which patients are not eligible for treatment?

Exclusion criteria are in accordance with those for patients with hyperinflammation.

*Recommendations for patients with immunosuppression*

Clinical phenotype:

- Increased susceptibility to secondary infections in the blood and lungs [96]
- Persistence of septic shock with ≥2 organ dysfunctions after initial resuscitation treatment *(Expert Opinion)*
- Persistent immunosuppression determined by e.g. high PD-1, lymphopenia, low IgM levels, low HLA-DR expression on monocytes, expansion of MDSCs [111, 124–126] *(Expert Opinion)*
- Clinical examples: nosocomial infections, secondary fungal infections (e.g. Aspergillosis), viral reactivation, insufficient clearance of primary infective focus, multi-morbid elderly patient (diabetes mellitus, liver disease, renal insufficiency, malnutrition), patients with viral (co-)reactivation [110]

Timing:

- Exact recommendation is difficult, but suggest that patients with severe sepsis or septic shock require rapid infusions to counteract the potential downstream effects *(Expert Opinion)*

Dosage:

- Total dose at least: 0.72 g/kg
- Continuous maintenance rate of 0.2 mL (10 mg)/kg/hour for 72 hours; IgM levels should be monitored if possible, and if no increase is observed, treatment should be prolonged for at least 2 additional days *(Expert Opinion)*

Exclusion criteria:

- Standing DNR order or limitation of therapy, incurable metastatic malignant disease, unstable hematological malignancies

### Monitoring immunoglobulin levels during therapy

Understanding when to stop therapy is important to prevent overtreatment and for economic reasons. We believe that there is a synergistic impact of simultaneously low levels of IgGAM during sepsis, and we suggest that immunoglobulin level kinetics may be a suitable marker for monitoring and modifying treatment, although we emphasize that the required minimum levels of circulating IgM, IgG and IgA are unclear at this point and further data are required to determine the scale of changes in immunoglobulins after treatment (Table 1). Based on current experience, we propose that patients with pathologically low levels of IgM should reach a sustained elevation to values > 80 mg/dL. Serial measurements of IgA, IgG, and IgM could help to correlate supplementation with outcome and important secondary endpoints in the future and define the optimal immunoglobulin levels required [21, 24, 94].

It is also important to consider that immunoglobin levels may be influenced by other treatment interventions such as fresh frozen plasma (which increases IgM) and rituximab (which significantly lowers IgM). Another consideration is the accumulation of IgM and IgA among chronic kidney disease patients; due to their high molecular weights, IgM and IgA are not removed by conventional renal replacement treatments such as continuous veno-venous hemodialysis and diafiltration (CVVH and CVVHD, respectively). Additionally, the possible effect of other blood purification techniques on immunoglobulin levels is not yet well established [127]. Even though not commonly used in septic shock patients, plasma exchange methods are able to remove both IgM and IgA due to the high sieving coefficient of the membranes used in this technique [128].

#### A novel situation in COVID-19

In 2019, severe acute respiratory syndrome coronavirus-2 (SARS-CoV-2) caused a pandemic with an unprecedented global crisis. Current data suggest a link between the severity of coronavirus disease 2019 (COVID-19), viral production, and the severe dysregulation of the inflammatory immune reaction (‘cytokine storm’). It is still unclear, however, which molecular mechanisms trigger the onset of the immune disbalance and why it can rapidly progress to multiorgan dysfunction or acute respiratory distress syndrome (ARDS) with a fatal outcome in a considerable subset of patients [129, 130].

Clinical observation of fatal courses of COVID-19 often includes severe ARDS, which is caused by alveolar injury and multiple organ failure—both of which are associated with hyperinflammation and cytokinemia [131]. Both mild and severe/fatal cases display changes in cytokine production, particularly IL-1β, IL-1ra, IL-6, IL-10, TNF-α, GM-CSF, IL-17, and pathological shifts of circulating leukocyte subsets [132, 133]. This leads to the disturbed development of protective immunity against the infection. The most severe complications of COVID-19 include sepsis-like inflammation, pulmonary or cardiovascular complications, and coagulopathy [134–136].

As discussed above, the innate host immune system is activated in response to the virus to limit infection. Subsequently, the adaptive immune system develops specific immunoglobulins and activates T-cells in direct response to the virus. If this inflammation is unmodulated or excessive, there is a risk of chronic hyperinflammation resulting in functional inhibition of the adaptive immune system. In addition to virus-induced lymphopenia, this can result in progressive tissue and organ damage, and failure of the adaptive immune system to develop functional immunoglobulins and clear the pathogen [137]. In theory, the use of IgGAM in patients showing signs of both hyper- and hypoinflammation could therefore be an effective therapeutic strategy. Investigations with Ig M- and IgA-enriched immunoglobulin are on the way. The beneficial use was reported in a first case report in a patient with hyperinflammation [138].

#### Unwanted side effects and adverse reactions

The use of IVIG as supportive therapy in sepsis is not entirely without controversy or risk. In some patients, serious adverse reactions consist of the development of a hyperviscosity syndrome with thromboembolic events. Further, acute renal failure has been observed, which was presumably associated with stabilizers contained in the IVIG preparations. IVIG-associated renal failure is most common in patients with pre-existing conditions such as renal impairment, diabetes mellitus, advanced age, volume depletion, or concomitant use of other substances known to cause renal toxicity [139]. However, most of these potential complications can be prevented by taking appropriate countermeasures. For example, slow infusion rates and adequate hydration may help to avoid renal failure as well as thromboembolic events [140].

## Conclusions and potential future research

It is evident that there are still many uncertainties associated with the diagnosis and particularly the management of different types of patients with sepsis. Research to effectively phenotype and characterize patient populations which correlate with a propensity to respond to treatment will be essential in tailoring management to the individual patient [141]. In this article we have described two distinct populations we believe would most benefit from therapy with IgM- and IgA-enriched immunoglobulins. For patients with hyperinflammation, clinical phenotypes are better recognized compared with patients with immunosuppression. Whilst there are more tools and biomarkers available for diagnosing patients with hyperinflammation compared to patients with immunosuppression, universally valid thresholds for these biomarkers (PCT, IL-6 and CRP) need to be elucidated. We also suggest that the timing of therapy with IgM- and IGA enriched immunoglobulin may be critical for patients with hyperinflammation, with early treatment showing the greatest benefit. These patients may further benefit from an initial bolus of Pentaglobin followed by a maintenance dose. However, further clinical or real-world evidence is required to make decisive recommendations regarding timing and dosage of treatment.

Among patients with immunosuppression, relevant biomarkers are largely debated, and research into developing technologies or identifying easily measured markers would be very valuable. Timing and dosage of therapy with IgM- and IgA-enriched immunoglobulins among immunosuppressed patients with chronic critical illness is also uncertain since the only available evidence is taken from patients with sepsis or septic shock. Therefore, clinical trials to identify optimal target parameters are critical to define the appropriate therapy parameters for this patient population.

For both patient populations, deciding when to discontinue therapy is also important. Pharmacokinetic and dose-response studies that monitor IgM, IgA and IgG levels in patients on IgM-immunoglobulin therapy should be carried out. It may also be of interest to study the impact of treatment with IgM- and IgA-enriched immunoglobulins on sepsis-related complications including critical illness polyneuropathy.

Within this manuscript, we characterized two different phenotypes of patients with sepsis and/or septic shock. This segregation is supported at a genomic level by a recent cohort study looking at the transcriptome variation of a large group of patients with severe community-acquired pneumonia (sCAP). Two distinct sepsis response signatures (SRS 1 and SRS 2) were identified, of which one group (SRS 1) showed clear signs of relative immunosuppression, endotoxin tolerance, and T-cell exhaustion, and was accompanied by a significantly worse outcome [142]. We hypothesize that patients exhibiting this phenotype might be likely to benefit from the administration of IgGAM. We acknowledge, however, that clinical reality currently excludes genetic/transcriptomic analyses, and that there is considerable overlap between these types of host response.

Clearly, more evidence is required to determine several specific aspects of treatment with IgM- and IgA-enriched immunoglobulins in patients with hyperinflammation and immunosuppression [143]. We conclude that, compared with IgG-only formulations which did not improve survival rates in patients with sepsis [47–49], treatment with IgM- and IgA-enriched immunoglobulins is very likely associated with a reduction in mortality and morbidity in terms of length of ventilatory support, length of ICU stay, and risk of secondary infectious complications [50–52, 71].

## Supplementary information

**Additional file 1: Appendix S1.** Participants at the Expert Meeting, which took place at the 39th *International Symposium on Intensive Care and Emergency Medicine* (ISICEM) congress in Brussels, Belgium in March 2019.

## Data Availability

Not applicable

## Abbreviations

APC: Antigen-presenting cells
ARDS: Acute respiratory distress syndrome, CI confidence interval
CMV: Cytomegalovirus
CRP: C-reactive protein
DAMP: Damage-associated molecular pattern
CVVH: Continuous veno-venous hemodialysis
CVVHD: Continuous veno-venous hemodialysis and diafiltration
DNA: Deoxyribose nucleic acid
DNR: Do not resuscitate
HR: Hazard ratio
HLA-DR: Human leukocyte antigen–antigen D related
HSV: Herpes simplex virus
ICU: Intensive care unit
IFN: Interferon
IgA: Immunoglobulin A
IgG: Immunoglobulin G
IgM: Immunoglobulin M
IL: Interleukin
IVIG: Intravenous immunoglobulin
MDR: Multidrug-resistant
MDSC: Myeloid-derived suppressor cell
NET: Neutrophil extracellular traps
NF-kB: Nuclear factor kappa-light-chain-enhancer of activated B cells
M-H: Mantel–Haenszel
OR: Odds ratio
PAMP: Pathogen-associated molecular pattern
PCT: Procalcitonin
PD-1: Programmed death protein 1
PD-L1: Programmed death ligand 1
PIRO: Predisposition: insult/infection: response and organ dysfunction
sCAP: Severe community-acquired pneumonia
SMD: Standard mean difference
SmPC: Summary of product characteristics
TGF β: Transforming growth factor-β
TLR: Toll-like receptor
TNF-ɑ: Tumor necrosis factor α
TO: Torino
Treg: Regulatory T
