# Supplementary material for: Best-practice IgM- and IgA-enriched immunoglobulin use in patients with sepsis
Source: Ann Intensive Care. 2020 Oct 7;10:132. doi: 10.1186/s13613-020-00740-1 (PMC7538847; doi:10.1186/s13613-020-00740-1)
Supplement: Supplementary file 1 — Additional file 1: Appendix S1. Participants at the Expert Meeting, which took place at the 39th International Symposium on Intensive Care and Emergency Medicine (ISICEM) congress in Brussels, Belgium in March 2019. [file 13613_2020_740_MOESM1_ESM.docx]

**Appendix 1.** Participants at the Expert Meeting, which took place at the 39^th^ International Symposium on Intensive Care and Emergency Medicine (ISICEM) congress in Brussels, Belgium in March 2019

| Prof. Giorgio Berlot | University of Trieste, Italy |
| --- | --- |
| Prof. Massimo Girardis | University of Modena, Italy |
| Prof. Detlef Kindgen-Milles | University Hospital Düsseldorf, Germany |
| Prof. Zsolt Molnár | University of Szeged, Hungary |
| Prof. Eckhard Müller | Evangelical Hospital Herne, Germany |
| Dr. Axel Nierhaus (Chair)  Biotest AG employees | University Medical Center Hamburg, Germany  Biotest AG |
